# Supplementary material for: The neural transdifferentiation potential of bone marrow mesenchymal stem cells
Source: PLoS One. 2026 Apr 20;21(4):e0347119. doi: 10.1371/journal.pone.0347119 (PMC13094960; doi:10.1371/journal.pone.0347119)
Supplement: Attachment — Submitted filename: Response to Reviewers.docx [file pone.0347119.s008.docx]

1. *“Please ensure that your manuscript meets PLOS ONE's style requirements, including those for file naming. The PLOS ONE style templates can be found at https://journals.plos.org/plosone/s/file?id=wjVg/PLOSOne_formatting_sample_main_body.pdf and https://journals.plos.org/plosone/s/file?id=ba62/PLOSOne_formatting_sample_title_authors_affiliations.pdf”*

This has been updated.

2. *“Please ensure you have included the registration number for the clinical trial referenced in the manuscript.”*

This is included in the manuscript.

3. *“Please note that PLOS One has specific guidelines on code sharing for submissions in which author-generated code underpins the findings in the manuscript. In these cases, we expect all author-generated code to be made available without restrictions upon publication of the work. Please review our guidelines at https://journals.plos.org/plosone/s/materials-and-software-sharing#loc-sharing-code and ensure that your code is shared in a way that follows best practice and facilitates reproducibility and reuse.”*

All code available.

4. *“We note that the grant information you provided in the ‘Funding Information’ and ‘Financial Disclosure’ sections do not match.*

*When you resubmit, please ensure that you provide the correct grant numbers for the awards you received for your study in the ‘Funding Information’ section.”*

This has been added.

5. *“Thank you for stating the following financial disclosure:*

*“M.Y.: PhD stipend from Helse Vest, Bergen, Norway”*

*Please state what role the funders took in the study. If the funders had no role, please state: "The funders had no role in study design, data collection and analysis, decision to publish, or preparation of the manuscript."*

*If this statement is not correct you must amend it as needed.*

*Please include this amended Role of Funder statement in your cover letter; we will change the online submission form on your behalf.”*

This has been added to the cover letter.

6. *“We note that your Data Availability Statement is currently as follows: “All relevant data are within the manuscript and its Supporting Information files. Patient information is limited to what is already included in the method section of the manuscript.*

*Please confirm at this time whether or not your submission contains all raw data required to replicate the results of your study. Authors must share the “minimal data set” for their submission. PLOS defines the minimal data set to consist of the data required to replicate all study findings reported in the article, as well as related metadata and methods (https://journals.plos.org/plosone/s/data-availability#loc-minimal-data-set-definition).*

*For example, authors should submit the following data:*

*- The values behind the means, standard deviations and other measures reported;*

*- The values used to build graphs;*

*- The points extracted from images for analysis.*

*Authors do not need to submit their entire data set if only a portion of the data was used in the reported study.*

*If your submission does not contain these data, please either upload them as Supporting Information files or deposit them to a stable, public repository and provide us with the relevant URLs, DOIs, or accession numbers. For a list of recommended repositories, please see https://journals.plos.org/plosone/s/recommended-repositories.*

*If there are ethical or legal restrictions on sharing a de-identified data set, please explain them in detail (e.g., data contain potentially sensitive information, data are owned by a third-party organization, etc.) and who has imposed them (e.g., an ethics committee). Please also provide contact information for a data access committee, ethics committee, or other institutional body to which data requests may be sent. If data are owned by a third party, please indicate how others may request data access.”*

Yes all raw data are now included.

7. *“If the reviewer comments include a recommendation to cite specific previously published works, please review and evaluate these publications to determine whether they are relevant and should be cited. There is no requirement to cite these works unless the editor has indicated otherwise.”*

No extra citations were recommended.

Additional Editor Comments (if provided):

The manuscript "The neural transdifferentiation potential of bone marrow mesenchymal stem cells" is an interesting investigation in the field of stem cell therapeutics. But it needs significant improvement before acceptance. So, revise the manuscript in the light of reviewers' comments.

Reviewer #1: The study by Ytterdal et al entitled “The neural transdifferentiation potential of bone marrow mesenchymal stem cells” examines the ability of mesenchymal stem cells (MSCs) to transdifferentiate to a neuro and glial lineages. The study addresses a decades-long controversy regarding the ability of MSCs to transdifferentiate from mesodermal to neuroectodermal lineage, a phenomenon suggested to be due to phenotypic plasticity and experimental artifact rather than true lineage conversion. While the current study offers some important insights in terms of timing and proteomic changes during transdifferentiation, there remains many deficits in the study including a lack of quantitation and statistical analysis that limit the overall interpretation that acquisition of a neural/glial phenotype by MSCs represents transdifferentiation.

1) “*Introduction lines 77-82: The authors cite 3 clinical trials using MSCs in MS. The first 2 trials represent IV administration of MSCs in RRMS patients. The limitations of the 2 studies should be noted, including IV administration on a RRMS patient population that already benefit from highly effective therapies thus limiting the need for MSCs in this population. A description of Ref 17 should also be included in this paragraph because it represents IT administration and reported efficacy in a progressive patient population.”*

We thank the reviewer for their thorough evaluation of the manuscript and for the constructive comments. The introduction lines 77-98 has been revised to note the limitations of the 2 intravenous MSC trials conducted in RRMS patients. We have also included a description of Ref 17, emphasizing its intrathecal delivery approach and its relevance to progressive MS.

2) “*Include additional demographics on the six donors including MS subtype, EDSS, disease duration, gender, and age.”*

We agree with this suggestion. Additional donor demographic information has been included in Table 1 in the manuscript, including MS subtype, gender, age, disease duration (years since the diagnosis) and EDSS for all seven donors. We have also included which donor MSCs that have been used for each experiment in the figure legends in the Results section.

3) “*Figure 1 is subjective and could easily represent a biased interpretation.
a. The undifferentiated MSCs which were repeatedly passaged over the 3 weeks are shown at subconfluence whereas the differentiated MSCs are confluent. The morphology of representative confluent MSCs should be used to compare.”*

We agree with the reviewer and have now included morphology of confluent undifferentiated MSCs to be able to compare the morphology with the transdifferentiating MSCs.

b. “*The advantage of using 6 separate MSC lines is to provide quantitative data. Any changes in morphology needs to be demonstrated for all 6 lines. The authors should apply high resolution imaging metrics using Image J or Cell Profiling to provide more robust and unbiased quantitation of basic morphological differences between MSCs, N- and G-MSCs from all 6 lines. At the very least, additional representative images of the other 5 lines should be included in the supplemental data. Figure legend should state that the images are representative.”*

The images of cultured MSCs were included for illustrative purposes, to demonstrate typical cell appearance under the different culture conditions and were not intended to support quantitative morphological analyses. Quantitative high-resolution image analysis across all donors was beyond the scope of the current work, which focused on functional and molecular characterization rather than detailed morphological phenotyping. To avoid any potential misinterpretation, we have clarified in the Fig 1 legend that the images shown are representative and are provided for illustrative purposes only. We did not have camera in the cell lab when we started these experiments, so we don’t have images of all donor MSCs.

c. “*The authors should address the morphological similarity between neural and glial differentiated cells despite claiming they represent different lineages. Other than being “different” from MSCs, please comment on how N or G MSC morphology compares to brain derived neural/glial cultures.”*

We revised the first paragraph of the Results section to address the morphological similarities and differences between G-MSC and N-MSC, compared to brain-derived neural and glial cultures.

We agree that the N-MSC and G-MSC show overlapping morphological features in vitro, indicating they are not fully transdifferentiated into neural and glial lineages. In our cultures, both N-MSCs and G-MSCs display elongated and aligned growth patterns, which is less complex than those observed in primary brain-derived neural or glial cultures. Importantly, the N- and G-MSCs generated in this study represent progenitor-like populations rather than fully mature neurons or oligodendrocytes. We do not expect them to exhibit complex and highly specialized morphologies as terminally differentiated neurons or oligodendrocytes derived from brain tissue. Therefore, lineage distinction in our study is not concluded from morphology alone, but rather from lineage-associated marker expression and proteomics as presented in the manuscript.

4) Figure 2 scratch assay:
a. “*Please justify the conclusion that the scratch assay is reflective of migratory capacity of MSCs. Clearly the proliferation rate of N- and G-MSCs is reduced and thus reflected in the reduced rate of closure in the scratch assay. The limitation of this assay in terms of assessing migratory capacity and its relationship to proliferation should be clearly delineated.”*

A limitation of the migration assay is that there always will be a component of proliferation. However, if the reviewer would agree with our reasoning, there is a large difference in migration capacity as well: In the proliferation assay (Fig 3), we observe a ~25% difference in proliferation between MSCs and the transdifferentiating cells at 48 hours (43% vs 32% confluency). In the migration assay (Fig 2), we observe a 35% difference already after 24 hours (78% vs 51% confluency), which indicates that a profound part of the confluency is due to migration. We agree that this is not sufficiently explained in the text and have thus updated it in the discussion.

b. “*The discussion point line 454 “decreased migratory capacity could hinder homing to CNS lesions” should be removed or revised since migration in this context refers to specific chemotactic responses that are typically measured in a trans well assay.”*

We agree with the reviewer’s point. The statement suggesting that decreased migratory capacity could hinder homing to CNS lesions has been removed from the Discussion section to avoid overinterpretation beyond the scope of the migration assay used in this study.

c. “*The legend states average and SE from 3 different experiments. Please clarify whether the graph represents all 6 cell lines that were tested in 3 different experiments and how the average and SE were determined. Data from all 6 cells lines needs to be included along with statistical analysis to support any differences between MSCs and N/G-MSCs.”*

The data shown in the graph represent results obtained from three donor MSCs, generated from three independent experiments. Average values were calculated using data pooled from all three donor MSCs across the independent experiments. Statistical analyses comparing MSC with N- and G- MSCs were performed using the full dataset, as described in the revised Statistical Analysis section of the Methods. We have revised the legend in Fig 2, Methods and Results sections to clearly describe the experimental design, number of donor MSCs, and statistical approach used.

5) “*Figure 3 proliferation: See point 4c above regarding data representing all 6 cells lines and statistical analysis.”*

Three MSC donors were used in the proliferation assay and three independent experiments. We have clarified this and included statistical analysis and data representing three donor MSCs in the Methods, Results and Fig 3 legend in the manuscript.

6) “*Figure 4 needs to include quantitative analysis of immunostaining in all 6 cell lines to demonstrate reproducibility. The figure showing a representative image of each marker is meaningless without quantitative analysis and statistics. There appears to be more GFAP staining in N/G MSCs but there are also more cells in the images. This reflects the fact that immunofluorescence is not ideal to quantitate protein expression and should be supported by a more quantitative method (Western blotting) in all 6 lines.”*

We have now included a quantitative analysis of the immunostaining shown in Fig 4. Quantification was performed from two independent transdifferentiation experiments using MSCs from two different donors.

To measure the fluorescence intensity, 5 randomly selected fields (1000 µm x 1000 µm) for each coverslip with cells were analyzed in the software Qupath. This resulted in 10 measurements per marker and condition.

To account for differences in cell density between conditions, fluorescence data were normalized to undifferentiated MSCs, which were set to 100%, and the graph in Fig 4 therefore represent relative percentages of marker-positive cells normalized to MSC controls. Statistical comparisons between MSCs and N-MSCs, as well as MSCs and G-MSCs, were performed using Welch’s t-test. Mean values with 95% confidence intervals are now shown, and statistically significant differences or non-significant are indicated in the graphs. This has now been revised in the Methods and Results sections.

7) “*Figure 5, the PCA plot appears to represent 2 samples of MSCs, 2 samples of N and G MSCs at 7 days, and 3 samples of N and G MSCs at 14 and 21 days. Please clarify whether these are representative of only 1 cell line in duplicate/triplicate or 3 cell lines? Please clarify whether or not proteomics was performed in all 6 lines.”*

Proteomics were performed on MSCs derived from six donors. However, only one donor had proteomic data available at all three transdifferentiation time points (7, 14, and 21 days). To allow a direct comparison of proteomic changes across transdifferentiation stages, the PCA plot in Fig 5 was therefore generated using samples from this single donor only.

Due to instrument capacity limitations (a maximum of 18 samples per mass spectrometry run), samples at day 7 were analyzed in duplicate, while samples at days 14 and 21 were analyzed in triplicate, enabling inclusion of all time points within a single run. This has now been clarified in the Results and Fig 5 legend.

8) Figure 6
a. “*please clarify why there are 7-9 biological replicates when the methods states 6 separate cell lines and the PCA plot only shows 2-3.”*

The heatmap in Fig 6 includes data from six MSC donors. One donor is represented twice, as this donor was analyzed in two separate experimental batches performed a year apart, using the same transdifferentiation culture condition. These two samples are therefore shown separately in the heatmap. This clarification has now been added to the Methods section and to the Fig 6 legend.

b. “*What were the criteria for selecting the markers represented in the heatmap?”*

The markers included in the heatmap were selected based on a literature review of proteins commonly used to characterize MSCs, neural and glial lineage-associated phenotypes, and cell proliferation or progenitor states. Selection criteria included, established relevance to MSC identity or neural/glial differentiation and consistent detection across the proteomic dataset.

c. “*The proteomic data should be analyzed by performing unbiased enrichment analysis to identify pathways that are altered. Without pathway analysis, it’s not clear that the markers as shown were cherry picked or are meaningful representatives of cell proliferation or differentiation towards a certain lineage.”*

To address the concern regarding potential selection bias and to provide an unbiased overview of the proteomic changes, we have now performed a KEGG pathway enrichment analysis based on the full proteomic dataset. The results are now included in the Results section and presented in a new figure (Fig 7). The analysis is also included in the Method section.

9) Figures 7, 8 and 9
a. “*The markers in these figures were from a predefined panel. Please explain the criteria for selection of the panel. Why are some of the markers not included in Figure 6?”*

To avoid confusion regarding donor representation and marker selection, we have removed Fig 7, 8, 9 and 10. These figures were based on data from a single donor with data from different transdifferentiation time, 7, 14 and 21 days. Instead, we now present all relevant proteomic data in the heatmap and KEGG-pathway which includes proteomics from six donors after 21 days of transdifferentiation time. This approach provides a clear and more comprehensive overview of marker expression across donors.

b. “*Clarify, again, whether the averages in the graphs represent 1 or 3 or 6 separate cell lines (see comment to Figure 5).”*

These figures have now been removed.

c. “*If not representative of all 6 cell lines, then additional protein quantitation should be performed (i.e. western blot) to confirm these protein changes in all 6 lines.”*

These figures have now been removed.

d. “*Please perform statistical analysis on graphs in figures 7, 8 and 9.”*

These figures have now been removed.

10) “*Figure 9, the oligodendroglial markers chosen do not accurately represent oligodendrocyte lineages. PDGFA is not typically expressed in oligodendrocytes as it is the ligand for oligo marker and receptor PDGFRA. The authors should show more typical markers of oligodendrocytes such as olig1 and olig2.”*

To avoid confusion, we have removed this figure and PDGFRa expression is shown in the heatmap in Fig 6.

11) Discussion
a. “*line 388 concludes that the expression patterns showed high consistency with minimal donor to donor variability. This statement is not supported by the data in all 6 cell lines as outlined in the above comments.”*

We have revised the text to avoid overinterpretation about expression patterns and minimal donor to donor variability. The statement has been modified to indicate that the expression patterns in the heatmap show generally consistent trends across donors, whit no obvious donor-specific outliers, rather than concluding minimal donor to donor variability.

b. “*Line 433, when discussing a more CNS-relevant secretory profile, only one of the factors (PDGFA) is secreted the others are cytoplasmic or membrane associated. Please revise this paragraph to clarify how these specific proteins might be related to improved trophic support of differentiated MSCs.”*

We have revised the paragraph in the discussion of the manuscript.

12) “*A major question regarding transdifferentiation is whether or not it represents an in vitro artifact due to stress caused by altered culture conditions. StemPro MSC medium contains a growth supplement (serum alternative). How much of the results shown here in terms of proliferation, ‘migration’, and expression of proliferation markers are due to removal of the growth supplement when culturing in StemPro NSC media? Furthermore, is the N or G phenotype reversible, meaning that after 3 weeks if you put the cells back in StemPro MSC do they resume proliferation? An even more important question is whether or not mesodermal differentiation (adipocytes, osteocytes, chondrocytes) is lost after N or G differentiation. Without additional experiments to answer these questions, the discussion needs to include a more balanced and tempered interpretation of the current findings regarding transdifferentiation.”*

It seems unlikely that the transdifferentiating of MSCs is due to stress because we show a higher expression of several neural and glial markers. We have now clarified in the Methods section that both the StemPro MSC medium and StemPro NSC medium include Supplement and thus do not impact the proliferation and migration experiments.

13) “*Address why G and N MSCs have similar morphology and protein expression despite exposure to different growth factors. The appropriate control would be StemPro NSC media without growth factors to show that the growth factors are indeed promoting differentiation into glial and neural lineages. Without this experiment, the possible alternative interpretations of this data should be included.”*

Neural and glial MSCs displayed overlapping morphology and partially overlapping protein expression profiles despite exposure to different growth factors. This is not unexpected, as both conditions are designed to promote early neural or glial progenitor-like states rather than terminal neuronal or glial differentiation. At these progenitor stages, cells share morphological features, while lineage-specific differences are primarily reflected at the molecular level rather than the morphology.

The limitation that we did not include an additional control using StemPro NSC media without growth factors to directly measure the contribution of growth factors to lineage specification has now been added in the Discussion section.

*14) “Include a more thorough discussion on the controversy around transdifferentiation of MSCs and the limitations of the current study. Multiple references from the 2000’s initiated the controversy underlying whether transdifferentiation occurs or is an in vitro artifact. Those references are omitted here but serve important perspective on the topic. Please discuss the limitations of the current study including the lack of functional data, lack of evidence of terminal differentiation, and lack of differentiation controls for proliferative effects of the culture media as outlined above. Furthermore, the overall lack of evidence in the literature supporting in vivo differentiation of MSCs or Neural MSCs into neural lineages upon transplantation is a major limitation to the interpretation that neural differentiation is relevant to the regenerative capacity of these cells.”*

The debate about transdifferentiation of MSCs have been included in the Discussion section of the manuscript. Regarding the comment about lack of evidence of terminal differentiation, our goal was not to achieve terminal differentiation of MSCs, rather guide the MSCs in an early neural/glial progenitor state. This is now revised in the Discussion section and limitations of the study are included.

Reviewer #2: The manuscript titled “The neural transdifferentiation potential of bone marrow mesenchymal stem cells” reports original research investigating the ability of MSCs from multiple sclerosis (MS) patients to acquire neural and glial-like characteristics in vitro. The study is well designed, technically sound, and supported by detailed proteomic analysis that enhances its scientific depth. The findings are consistent and reproducible, indicating early neuro-glial lineage commitment. Overall, the paper is clearly written and contributes meaningful molecular insight into MSC biology. However, several issues concerning methodological transparency, statistical rigor, and ethical reporting should be addressed before it can be considered for publication. Please find the detailed comments below.

Major Comments
1- “*The introduction does not sufficiently explain the scientific reasoning for selecting MSCs from MS patients rather than healthy donors. The rationale should clarify whether the intent was to model disease-specific MSC behavior, to evaluate the feasibility of autologous transplantation in MS, or to test whether the disease state influences differentiation potential. Without this context, the choice of donor source appears arbitrary and limits interpretation of the findings.”*

The rationale for using MSCs derived from MS patients has now been clarified in the Introduction of the manuscript. Most clinical studies investigating MSC-based therapies for MS rely on autologous cell sources. Therefore, we study MSCs derived directly from MS patients to generate data that are directly applicable to autologous transplantation strategies in MS.

2- “*A section describing statistical methodology is missing. The results include qualitative claims of “significant” differences in migration and proliferation, but no information is provided on statistical tests, sample size, or p-values.”*

We have now included a dedicated Statistical Analysis subsection to the Methods. All quantitative comparisons of migration and proliferation were analyzed using linear mixed-effects models, which accounts for both fixed effects of experimental condition and random effects associated with biological replicates. Sample sized, model structure and significance criteria are now described, and exact p-values are reported in the Results where applicable.

3- “*The fluorescence images are interpreted qualitatively. If possible, quantitative analysis (e.g., mean fluorescence intensity or proportion of marker-positive cells across replicates) should be included.”*

We have now included a quantitative analysis of the immunostaining shown in Fig 4. Quantification was performed from two independent transdifferentiation experiments using MSCs from two different donors.

To measure the fluorescence intensity, 5 randomly selected fields (1000 µm x 1000 µm) for each coverslip with cells were analyzed in the software Qupath. This resulted in 10 measurements per marker and condition.

To account for differences in cell density between conditions, fluorescence data were normalized to undifferentiated MSCs, which were set to 100%, and the graph in Fig 4 therefore represent relative percentages of marker-positive cells normalized to MSC controls. Statistical comparisons between MSCs and N-MSCs, as well as MSCs and G-MSCs, were performed using Welch’s t-test. Mean values with 95% confidence intervals are now shown, and statistically significant differences or non-significant are indicated in the graphs. This has now been revised in the Methods and Results sections.

4- “*More detailed information about the six MS donors is needed, including sex distribution, MS subtype (progressive vs. relapsing), disease duration, and treatment history. These factors are relevant for assessing potential biological variability and for enabling reproducibility.”*

Additional donor demographic information has been included in Table 1 in the manuscript, including MS subtype, gender, age, disease duration (years since the diagnosis) and EDSS for all seven donors. We have also included which donor MSCs that have been used for each experiment in the figure legends in the Results section.

5- “*The manuscript lists “N/A” under Ethics Statement, although human-derived cells were used. Please revise to include the appropriate ethical approval (REK#159326) and confirm that written informed consent was obtained under the SMART-MS protocol.”*

We have now included the appropriate ethical approval under Ethics Statement.

6- “*The proteomic dataset is extensive, but the analysis is limited to marker-level observations. Incorporating pathway-level bioinformatic analyses (e.g., Gene Ontology or KEGG enrichment) would help identify broader biological processes and signaling pathways associated with transdifferentiation. This can be performed using the existing dataset and would considerably enrich the study’s interpretive depth.”*

To address the concern regarding potential selection bias and to provide an unbiased overview of the proteomic changes, we have now performed a KEGG pathway enrichment analysis based on the full proteomic dataset. The results are now included in the Results section and presented in a new figure (Fig 7). The analysis is also included in the Method section.

7- “*Terminology: “Transdifferentiation”. Since functional neural or glial activity was not demonstrated, it would be more accurate to refer to the observed changes as lineage priming or phenotypic conversion rather than full transdifferentiation.”*

We agree with the reviewer that functional neuronal or glial activity was not demonstrated in this study and that the observed changes do not represent terminal neural or glial differentiation. To address this, we have clarified our terminology in the Introduction section.

Specifically, we define transdifferentiating MSCs as MSCs directed to neural or glial lineages, characterized by progenitor-associated molecular and pathway-level changes rather than mature neural or glial functionality.

Minor Comments
1- “*Several typographical errors should be corrected (e.g., “trans-differentiated,” “neurodegeneratice”).”*

All identified typographical errors, including corrections of terms such as “trans-differentiated” and “neurodegeneratice”, have been corrected throughout the manuscript.

2- “*Confirm that data files in the Supporting Information fully correspond to the figures and text.”*

We confirm that all data files provided in the Supporting Information fully correspond to the figures and descriptions presented in the main text. The Supporting Information has been carefully reviewed to ensure consistency with the figures, legends, and results reported in the manuscript.
