# Supplementary material for: The neural transdifferentiation potential of bone marrow mesenchymal stem cells
Source: PLoS One. 2026 Apr 20;21(4):e0347119. doi: 10.1371/journal.pone.0347119 (PMC13094960; doi:10.1371/journal.pone.0347119)
Supplement: Attachment — Submitted filename: Response to Reviewers 20.03.docx [file pone.0347119.s009.docx]

*Journal Requirements:*

Journal Requirements:

1. «*If the reviewer comments include a recommendation to cite specific previously published works, please review and evaluate these publications to determine whether they are relevant and should be cited. There is no requirement to cite these works unless the editor has indicated otherwise. »*

There was no recommendation to cite specific published work.

*2. Please review your reference list to ensure that it is complete and correct. If you have cited papers that have been retracted, please include the rationale for doing so in the manuscript text, or remove these references and replace them with relevant current references. Any changes to the reference list should be mentioned in the rebuttal letter that accompanies your revised manuscript. If you need to cite a retracted article, indicate the article’s retracted status in the References list and also include a citation and full reference for the retraction notice.*

We have reviewed the reference list, no retracted articles were identified among the cited references, and therefore no changes to the reference list were required.

Additional Editor Comments:

1. *«The newly added graphs in Figure 4 are very small and difficult to read. Please revise the figure to improve clarity and readability. »*

Thank you for this important comment. The newly added graphs in Figure 4 have now been revised to improve clarity and readability.

*2. «Please comment on whether SOX2, PDGF, and NEFL were also observed to be upregulated in the proteomic screen. »*

Among these markers PDGFA was upregulated in the proteomic dataset. For inclusion in the proteomic heatmap, proteins were required to be consistently detected across all samples from all mass spectrometry batches. NEFL was detected only in one batch (where it was upregulated in neural-MSCs) and was therefore excluded from further analysis. For SOX2, the identifications did not meet the predefined confidence thresholds required for reliable protein assignment and quantitative analysis.

Discrepancies between the mass spectrometry data and the immunostaining likely arise from methodological differences. Antibody-based immunostaining allows sensitive and specific detection of predefined targets, whereas mass spectrometry–based proteomics relies on the presence, abundance, and analytical detectability of protein-derived peptides.

*3. «Line 448: The sentence beginning with “After 3 weeks of …” is incomplete, as the word “transdifferentiation” is missing. »*

The word transdifferentiation is now included.
